# Phenotypic and Genetic Diversity of *Aeromonas* Species Isolated from Fresh Water Lakes in Malaysia

**DOI:** 10.1371/journal.pone.0145933

**Published:** 2015-12-28

**Authors:** Wei Ching Khor, Suat Moi Puah, Jin Ai Mary Anne Tan, SD Puthucheary, Kek Heng Chua

**Affiliations:** 1 Department of Biomedical Science, Faculty of Medicine, University of Malaya, Kuala Lumpur, Malaysia; 2 Department of Medical Education, Research and Evaluation, Duke-NUS Medical School Singapore, Singapore; Auburn University, UNITED STATES

## Abstract

Gram-negative bacilli of the genus *Aeromonas* are primarily inhabitants of the aquatic environment. Humans acquire this organism from a wide range of food and water sources as well as during aquatic recreational activities. In the present study, the diversity and distribution of *Aeromonas* species from freshwater lakes in Malaysia was investigated using glycerophospholipid-cholesterol acyltransferase (*GCAT*) and RNA polymerase sigma-factor (*rpoD*) genes for speciation. A total of 122 possible *Aeromonas* strains were isolated and confirmed to genus level using the API20E system. The clonality of the isolates was investigated using ERIC-PCR and 20 duplicate isolates were excluded from the study. The specific *GCAT*-PCR identified all isolates as belonging to the genus *Aeromonas*, in agreement with the biochemical identification. A phylogenetic tree was constructed using the *rpoD* gene sequence and all 102 isolates were identified as: *A*. *veronii* 43%, *A*. *jandaei* 37%, *A*. *hydrophila* 6%, *A*. *caviae* 4%, *A*. *salmonicida* 2%, *A*. *media* 2%, *A*. *allosaccharophila* 1%, *A*. *dhakensis* 1% and *Aeromonas* spp. 4%. Twelve virulence genes were present in the following proportions—*exu* 96%, *ser* 93%, *aer* 87%, *fla* 83%, *enolase* 70%, *ela* 62%, *act* 54%, *aexT* 33%, *lip* 16%, *dam* 16%, *alt* 8% and *ast* 4%, and at least 2 of these genes were present in all 102 strains. The *ascV*, *aexU* and *hlyA* genes were not detected among the isolates. *A*. *hydrophila* was the main species containing virulence genes *alt* and *ast* either present alone or in combination. It is possible that different mechanisms may be used by each genospecies to demonstrate virulence. In summary, with the use of *GCAT* and *rpoD* genes, unambiguous identification of *Aeromonas* species is possible and provides valuable data on the phylogenetic diversity of the organism.

## Introduction

*Aeromonas* organisms are oxidase-positive, polar flagellated, non-sporulating facultative anaerobic rods [1]. These Gram-negative aeromonads are essentially ubiquitous in the microbial biosphere and found in almost every environmental niche, including aquatic habitats, fish, foods, domesticated pets, birds and soil. The aquatic environment is the natural habitat of aeromonads and they can be isolated from rivers, lakes, ponds, groundwater, surface water and chlorinated water. They are well known as causative agents of disease in fish, prawns, shrimps, oysters and other seafood [2]. *A*. *salmonicida* cause furunculosis and septicemia that result in huge economical loss in the fishing industry [3,4]. Disease may also be caused by the mesophilic *A*. *hydrophila* which has been linked to several epidemic outbreaks in the fishing industry [2,5]. In humans, aeromonads have been reported to be responsible for both gastrointestinal and extraintestinal infections particularly in immunocompromised patients [2,6,7]. Humans acquire aeromonads from a wide range of food and water [2]. Recreational activities such as boating, skiing, fishing and diving pose risks leading to infections [4,8].

The taxonomy of *Aeromonas* is in transition and presently this genus consists of 30 species [9]. Identification of *Aeromonas* to the species level can be difficult due to its complex phenotypic and genotypic heterogeneity [10]. The use of molecular approaches has led to a more refined identification that has revealed a number of discrepancies in the biochemical identification of this organism [11]. A molecular identification for *Aeromonas* species using *GCAT* and *rpoD* genes was reported by Puthucheary et al. [12]. *GCAT* is a highly conserved lipase gene present in practically all *Aeromonas* strains and a specific PCR probe was designed by Chacón et al. [13] that avoids confusion with other genera, such as *Vibrio* and *Plesiomonas*. The *rpoD* gene, a housekeeping gene, was reported to be an excellent tool for identification and for inferring the taxonomy of the genus *Aeromonas* [11]. Enterobacterial repetitive intergenic consensus (ERIC) sequences are short repetitive sequences in genomes of bacteria and the ERIC-PCR approach has been widely used for genomic fingerprinting of a broad range of bacterial species [14]. This method allows both phylogenetic inference and clonal differentiation of bacterial strains.

The production of virulence factors is essential for bacteria to establish infections and in *Aeromonas* a number of virulence genes have been described [15–18]. Pore-forming aerolysins *aer* and enterotoxins *act*, *alt* and *ast* are virulence determinants associated with gastroenteritis and diarrheal syndromes [19–21]. Other virulence factors described are extracellular lipases *lip*, *lipH3*, *pla* and *plc* that alter the host plasma membranes [22]. Type 3 secretion system (T3SS) effectors, *AexT* and *AexU* with the ability to cause host cell death have also been characterised [23,24]. Virulence in *Aeromonas* is a complex process and the detection of virulence factors is necessary in determining the potential pathogenicity and subsequent possible targets for vaccines. Hence the objectives of this study were (a) to isolate aeromonads from their natural aquatic habitat, (b) to investigate the clonality of the isolates using ERIC-PCR, (c) to identify and speciate these isolates using the *GCAT* and *rpoD* genes and (d) to screen for 15 virulence determinants.

## Materials and Methods

### Sample collection and bacterial isolation

Surface water samples were collected from 5 fresh water multi-purpose recreational lakes in Selangor, i.e., Tasik Aman (lake 1) (N 03.10261 °, E 101.62477 °), Tasik Taman Jaya (lake 2) (N 3.10418 °, E 101.64929 °), Tasik Varsiti Universiti Malaya (lake 3) (N 3.11941 °, E 101.65808 °) and two unnamed lakes in Rawang (lake 4 and lake 5) (N 03.36606 °, E 101.63717 ° and N 03.36752 °, E 101.63043 °). All samples were kept at 4°C and analysed within 30 hours of collection.

The samples were pre-filtered to remove residue and subsequently filtered through a 0.45 μm nitrocellulose membrane (Sartorius, Germany) using a vacuum system. The membranes were then suspended in broth and plated onto m-Aeromonas selective media (Biolife, Italia Srl) supplemented with ampicillin (10 mg/l) [25]. Yellow colonies on the agar plates due to dextrin fermentation, after 18–24 hours of incubation at 30°C were presumed to be *Aeromonas* species and tested with oxidase reagent (bioMérieux, France), checked for growth on MacConkey agar and 6.5% (w/v) NaCl-Luria Bertani (LB) broth. Oxidase-positive colonies growing on MacConkey agar but not in 6.5% NaCl-LB broth were further confirmed to genus level by the API 20E system (bioMérieux, France), then grown in LB broth, cryopreserved in 20% (v/v) glycerol at -80°C and maintained in LB agar and broth as working cultures.

### Bacterial DNA extraction

Genomic DNA extraction was carried out using GeneAll^®^ Exgene^™^ Cell SV DNA isolation kit (GeneAll Technology, Korea). Overnight cultures were pelleted, lysed using 20 mg/ml Proteinase K, passed through a spin column and washed with buffers and the purified DNA was subjected to concentration and standardisation for subsequent molecular analysis.

### Molecular identification

#### ERIC-PCR analysis

All isolates were subjected to ERIC-PCR fingerprinting using primers and PCR conditions as described previously [26]. The amplification products were electrophoresed in 1.5% (W/V) agarose gels containing ethidium bromide at 56V for 6 hours in Tris-borate-EDTA buffer. Gene Ruler 100 bp DNA Ladder Plus (Fermentas) was used as a molecular size reference. The electrophoresed gels were visualised using a UV light transilluminator. The digitised profiles were analysed by BioNumerics software, version 7.5 (Applied Maths, Belgium). Similarity between the fingerprints was calculated with the band-matching Dice coefficient. Cluster analysis was performed using the unweighted pair-group method with average linkages (UPGMA). Representative samples from each cluster were amplified using the *rpoD* gene and sent for sequencing for verification.

#### Genus identification

The *GCAT* gene was amplified using primer pair as reported previously [13]. Presence of this gene (237 bp) was visualised on 1.5% agarose gel stained with ethidium bromide.

#### Species identification

Strains with the *GCAT* gene were further subjected to *rpoD* gene sequencing whereby the 816 bp gene region was amplified by a touch-down PCR using a degenerate primer pair as described by Yamamoto et al. [27]. The amplified product of the *rpoD* gene was resolved on a 2% agarose gel, excised and subjected to purification using QIAquick Gel Extraction kit (Qiagen, Germany). The gel was dissolved completely to release the PCR product which was then passed through a spin column and washed with buffers for purification, then sent for sequencing using specific primers as reported by Yamamoto et al. [27]. The resulting DNA sequences were then compared with the GenBank database using Basic Local Alignment Search Tool (BLAST).

### Phylogenetic analysis

Phylogenetic data analysis using a partial nucleotide sequence (670 bp) of the *rpoD* gene was performed, the sequences (GenBank accession numbers: KT187565 to KT187686) were aligned using the ClustalW program and pairwise sequence identity matrices were calculated using the Bioedit software version 7.0.9 [28]. A phylogenetic tree was constructed based on neighbour-joining method via the MEGA 6 program with bootstrapping for 1000 replicates [29]. The genetic distances were also computed using Kimura's two-parameter model. The *rpoD* gene sequences of type strains representing all the known *Aeromonas* species were obtained from National Centre for Biotechnology Information (NCBI) database and used as reference gene sequences in the phylogenetic tree construction (Table 1). *Vibrio parahaemolyticus* ATCC 43996 (JQ015347.1) was included as an outgroup.

**Table 1:** Reference gene sequences used in the phylogenetic tree construction.

	Species	Accession no.
1	*A*. *allosaccharophila* CECT 4199	HQ442825
2	*A*. *australiensis* strain 266	FN773335
3	*A*. *bestiarum* CECT 4227	HQ442854
4	*A*. *bivalvium* CECT 7113	HQ442817
5	*A*. *carvenicola* CECT 7862	HQ442864
6	*A*. *caviae* CECT 838	HQ442790
7	*A*. *dhakensis* CECT 7289	HQ442798
8	*A*. *diversa* CECT 4254	HQ442805
9	*A*. *encheleia* CECT 4342	HQ442778
10	*A*. *eucrenophila* CECT 4224	HQ442770
11	*A*. *fluvialis* strain 717	FJ603453
12	*A*. *hydrophila* CECT 839	HQ442791
13	*A*. *jandaei* CECT 4228	HQ442840
14	*A*. *media* CECT 4232	HQ442785
15	*A*. *molluscorum* CECT 5864	HQ442812
16	*A*. *piscicola* CECT 7443	HQ442859
17	*A*. *popoffii* CECT 5176	HQ442853
18	*A*. *rivuli* DSM 22539	FJ969433
19	*A*. *salmonicida* CECT 894	HQ442843
20	*A*. *sanarellii* strain A2-67	FJ807275
21	*A*. *schubertii* CECT 4240	HQ442809
22	*A*. *simiae* CIP 107798	HQ442811
23	*A*. *sobria* CECT 4245	HQ442867
24	*A*. *taiwanensis* strain A2-50	FJ807271
25	*A*. *tecta* CECT 7082	HQ442762
26	*A*. *trota* CECT 4255	HQ442822
27	*A*. *veronii* CECT 4257	HQ442833

### Screening of virulence genes

Isolates identified as *Aeromonas* species by *rpoD* gene analysis were subjected to direct PCR to detect the existence of the following 15 known virulence genes encoding for, DNAse (*exu*), heat-labile cytotonic enterotoxin (*alt*), serine protease (*ser*), aerolysin/hemolysin (*aer*), cytotoxic enterotoxin (*act*), heat-stable cytotonic enterotoxin (*ast*), lipase (*lip*), flagellin (*fla*), elastase (*ela*), ADP-ribosyltransferase toxins (*aexT* and *aexU*), DNA adenine methyltransferase (*dam*), enolase (*enolase*), T3SS membrane component (*ascV*) and hemolysin (*hlyA*). Screening was performed using the PCR primers reported previously (Table 2) at annealing temperatures from 55 to 68°C. Two-tailed Fisher’s exact test was carried out to determine the presence of combination of virulence genes. The purified PCR products of the genes, detected by QIAquick gel extraction kit (Qiagen, Germany) were sent for sequencing for validation.

**Table 2:** Primers selected to detect virulence genes.

Gene	Primer sequence (5’ to 3’), F/R	Size (bp)	Reference
*exu*	(A/G)GACATGCACAACCTCTTCC/ GCTTGGTATTGCC(C/T)TGCAA(C/G)	323	[30]
*ser*	ACGGAGTGCGTTCTTCCTACTCCAG/ CCGTTCATCACACCGTTGTAGTCG	211	[31]
*aer*	CCTATGGCCTGAGCGAGAAG/ CCAGTTCCAGTCCCACCACT	431	[15]
*fla*	TCCAACCGTYTGACCTC/ GMYTGGTTGCGRATGGT	608	[18]
*act*	AGAAGGTGACCACCAAGAACA/ AACTGACATCGGCCTTGAACTC	232	[32]
*ela*	ACACGGTCAAGGAGATCAAC/ CGCTGGTGTTGGCCAGCAGG	513	[18]
*aexT*	CGTGGCCATCAAAGAGTGG/ GCAGCTGGCTCATCGCCTC	425	[33]
*lip*	ATCTTCTCCGACTGGTTCGG/ CCGTGCCAGGACTGGGTCTT	382	[18]
*alt*	AAAGCGTCTGACAGCGAAGT/ AGCGCATAGGCGTTCTCTT	320	[34]
*ast*	ATCGTCAGCGACAGCTTCTT/ CTCATCCCTTGGCTTGTTGT	504	[34]
*dam*	ATGAAAAAAACACGCGCTTTTTTAAAATGG/ TCAGCCGAGTGGCGCCAGTTCGGCGTCG	873	[16]
*enolase*	ATGTCCAAGATCGTTAAAGTGAT/ TTAAGCCTGGTTCTTCACTTCTT	1302	[35]
*ascV*	ATGAAGCCCGCTTCGCCTATCAA/ TCACAGGCAGACCCTTCCCAGC	2166	[36]
*aexU*	ATGCAGATTCAAACACATACCAGCGGC/ TTACAGATAGTCAGCCCCGACACCGAT	1539	[23]
*hlyA*	ATGAGTTTTGCCGATAGTTTATTTTTCCTGA/ TTACGATTCCTGAGCGGGCTTGTCGGCCGGCGTG	1320	[37]

## Results and Discussion

A total of 122 probable aeromonad strains were isolated from the 5 lakes in Selangor and identified as *Aeromonas* spp. using the API20E system, i.e. 27 from lake 1, 21 from lake 2, 15 from lake 3, 18 from lake 4 and 21 from lake 5.

**Fig 1:**
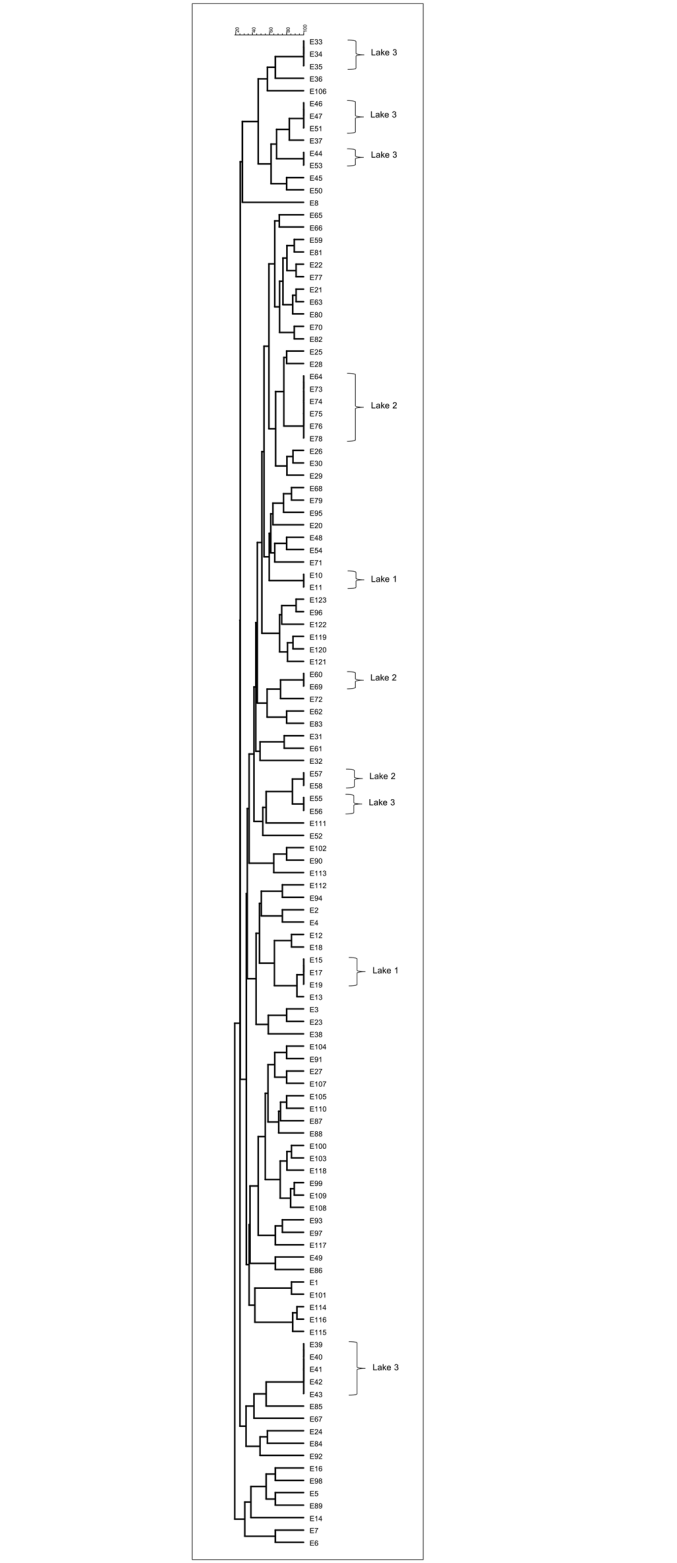
Dendrogram showing ERIC fingerprints of the 122 strains of *Aeromonas* isolates using Dice similarity coefficient and UPGMA cluster method.

ERIC fingerprinting revealed 10 clusters consisting of 30 isolates at the 100% similarity level (Fig 1). The strains within each cluster appeared to have identical fingerprints and were considered as belonging to the same clone. The verification test demonstrated identical *rpoD* gene sequences for the representative isolates of the same cluster thus confirming the clonality. Replicate isolates were excluded from the study and 102 samples were subjected to further analyses.

The genus-specific *GCAT* gene present in the 102 isolates, confirmed that all the environmental isolates belonged to the genus *Aeromonas*. Hence, the biochemical identification system API20E is in agreement with the *GCAT*-PCR in identifying *Aeromonas* to the genus level. But the API20E has limitations in accurately identifying *Aeromonas* species without additional biochemical, morphological and physiological tests, thus the necessity of molecular methods for a higher resolution identification of *Aeromonas* species.

**Fig 2:**
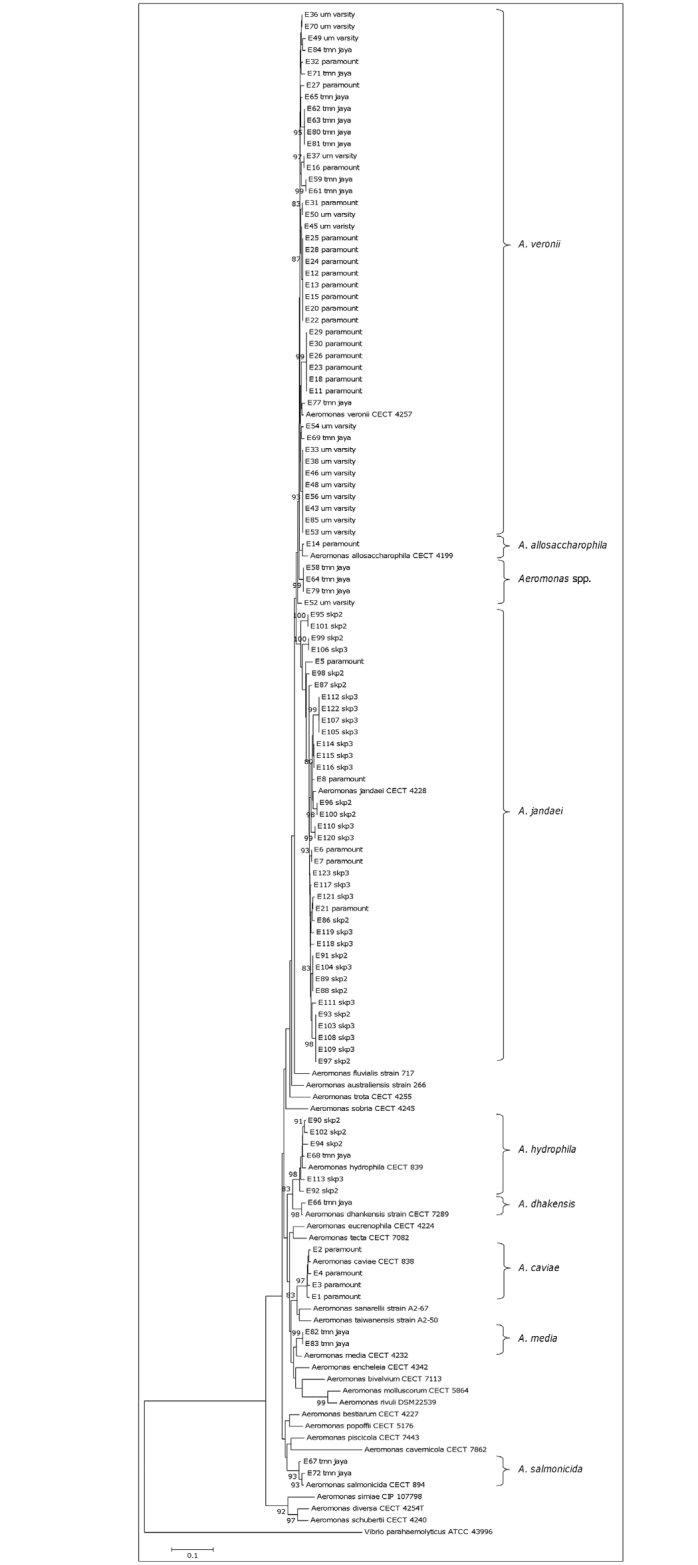
Phylogenetic tree of 102 *Aeromonas* and reference strains based on the *rpoD* gene sequences using neighbour-joining method with bootstrap replication of 1000.

Based on the constructed phylogenetic tree using the partial *rpoD* sequence, 98 isolates showed clustering with the reference strains of respective species and were identified as: *A*. *veronii*—44, *A*. *jandaei*—38, *A*. *hydrophila*—6, *A*. *caviae*—4, *A*. *salmonicida*—2, *A*. *media*—2, *A*. *allosaccharophila*—1 and *A*. *dhakensis*—1 (Fig 2). Four isolates were not grouped into any cluster with known species strain. They formed clusters branching close to *A*. *allosaccharophila* and *A*. *veronii*, suggesting that these isolates need further investigation using more than one target gene or other approaches. The 4 isolates were considered as “*Aeromonas* spp.” in this study. The pairwise sequence identity matrix using the *rpoD* gene revealed that intraspecies similarities for the isolates were 96.2–100% for *A*. *veronii*, 95.2–100% for *A*. *jandaei*, 97.3–98.6% for *A*. *hydrophila*, 97.1–99.4% for *A*. *caviae*, 98.0% for *A*. *salmonicida* and 100% for *A*. *media* and 96.7–100% for *Aeromonas* spp.

*A*. *veronii* was the most common species– 43% followed by *A*. *jandaei* 37%, *A*. *hydrophila* 6%, *A*. *caviae* 4%, *A*. *salmonicida* 2%, *A*. *media* 2%, *A*. *allosaccharophila* 1% and *A*. *dhakensis* 1% and *Aeromonas* spp. 4%. We found dominance of *A*. *veronii* in environmental samples [38,39]. *A*. *jandaei*–the second common species in this study, was reported to be associated with epizootic occurrence in farmed eels [40] and possible implications for clinical infections [41]. *A*. *salmonicida* is a predominant species related to fish and fresh water samples [2]. However, only 2 strains of *A*. *salmonicida* were detected in our study and this may be due to several factors such as geographical location, type of aquatic environment selected or the temperature and pH of the water as *A*. *salmonicida* is not able to grow at temperatures around 37°C [42,43]. This variance can also be related to different genes or different regions of the same gene being targeted for the bacterial identification.

Biochemically, all *Aeromonas* isolates were oxidase-positive and fermented glucose and mannose, but showed negative reactions for ornithine decarboxylase, hydrogen sulfide, urease, tryptophan deaminase and rhamnose fermentation. Most *A*. *jandaei* were citrate-positive whereas only half of *A*. *veronii* were able to use citrate as the sole carbon source. Thirty-four percent of *A*. *jandaei* fermented melibiose while none of the *A*. *veronii* strains showed fermentation. The findings were in agreement with Abbott et al., [44]. However, 7 (18%) *A*. *jandaei* strains were observed to be atypically sucrose-positive.

Multiple virulence genes were present in all the 102 *Aeromonas* strains and all contained at least 2 of the virulence genes but none possessed only 1 virulence gene (Table 3). A single *A*. *hydrophila* isolate from the lake 4 carried a complement of 12 of the 15 virulence genes. The *exu* gene was the most prevalent, being present in 96% of the isolates and widely distributed in all species, except of *A*. *media*, similar to the study by Chacón et al. [15]. Other virulence genes detected were *ser* 93%, *aer* 87%, *fla* 83%, *enolase* 70%, *ela* 62, *act* 54%, *aexT* 33%, *lip* 16%, *dam* 16%, *alt* 8% and *ast* 4%. The sequencing results were compared to the Genbank database using BLAST. The nucleotide BLAST homology search revealed high homologies (>90%) of the virulence gene PCR products with the deposited sequences in the database.

**Table 3:** Presence of multiple virulence genes in 102 *Aeromonas* isolates.

Species	Frequency of isolates harbouring the indicated number of virulence gene, %															
	1	2	3	4	5	6	7	8	9	10	11	12	13	14	15	Total
																(≥2)
*A*. *veronii*	-	-	-	3	11	16	9	5	-	-	-	-	-	-	-	44
(n = 44)				**7**	**25**	**36**	**20**	**11**								**100**
*A*. *jandaei*	-	-	2	2	6	21	6	1	-	-	-	-	-	-	-	38
(n = 38)			**5**	**5**	**16**	**55**	**16**	**3**								**100**
*A*. *hydrophila*	-	-	-	-	-	-	-	1	-	3	1	1	-	-	-	6
(n = 6)								**17**		**50**	**17**	**17**				**100**
*A*. *caviae*	-	-	-	-	2	1	-	1	-	-	-	-	-	-	-	4
(n = 4)					**50**	**25**		**25**								**100**
*A*.*salmonicida*	-	-	-	-	-	-	-	-	-	2	-	-	-	-	-	2
(n = 2)										**100**						**100**
*A*. *media*	-	-	-	1	1	-	-	-	-	-	-	-	-	-	-	2
(n = 2)				**50**	**50**											**100**
*A*. *allasaccharophila*	-	-	-	-	1	-	-	-	-	-	-	-	-	-	-	1
(n = 1)					**100**											**100**
*A*. *dhakensis*	-	-	-	-	-	-	-	-	1	-	-	-	-	-	-	1
(n = 1)									**100**							**100**
*Aeromonas* spp.	-	-	-	2	1	-	1	-	-	-	-	-	-	-	-	4
(n = 4)				**50**	**25**		**25**									**100**

Two-tailed Fisher’s exact test based on each virulence gene revealed statistically significant associations in *A*. *hydrophila* with *alt*, *ast*, *lip*, *aexT* and *dam*; *A*. *caviae* with *lip*; *A*. *veronii* with *act* and *aexT* and *A*. *salmonicida* with *dam* (Table 4). The serine protease gene was detected in all species (≥95%) with the exception of *A*. *caviae* (25%). The presence of *aer* gene was observed in *A*. *veronii* 94% and *A*. *hydrophila* 83%, but less frequently among *A*. *caviae* 25%, in agreement with Yousr et al. [45]. Inverse associations were observed in *A*. *jandaei* with the *lip*, *fla* and *aexT* genes and *A*. *veronii* with the *lip*, *ela* and *enolase* genes. None of the isolates demonstrated the presence of the *ascV*, *aexU* and *hlyA* genes. Khajanchi et al [46] reported that 30–47% of their isolates obtained from water samples were positive for the *ascV*, *aexU* and *hlyA* genes. This discordance can be explained by the fact that pathogenetic mechanisms of *Aeromonas* species may be different in different geographical locations. The discrepancies can also be related to the different dominant species of isolates in different studies. In their study, the predominant species was *A*. *hydrophila* (59.5%)—this species harboured the 3 genes. In contrast, there were only 6 strains of *A*. *hydrophila* (6%) in our study.

**Table 4:** Distribution of single and subsets of virulence genes in 102 *Aeromonas* isolates.

Single virulence gene	Frequency of isolates of the indicated species									
	*A*. *veronii* (n = 44)	*A*. *jandaei* (n = 38)	*A*. *hydrophila* (n = 6)	*A*. *caviae*(n = 4)	*A*. *salmonicida*(n = 2)	*A*. *media* (n = 2)	*A*. *allosaccharophila*(n = 1)	*A*. *dhakensis* (n = 1)	*Aeromonas* spp. (n = 4)	Total (n = 102)
*exu*	42	38	6	4	2	0	1	1	4	98
*alt*	0	0	**6** ^a^	0	1	0	0	1	0	8
*ser*	42	36	6	1	2	2	1	1	4	95
*aer*	41	35	5	1	2	0	0	1	4	89
*act*	**41** ^a^	6	2	2	2	1	0	0	1	55
*ast*	0	0	**4** ^a^	0	0	0	0	0	0	4
*lip*	0	2	**5** ^b^	**4** ^b^	2	2	0	1	0	16
*fla*	38	27	6	4	2	2	1	1	4	85
*ela*	11	37	6	4	2	2	0	1	0	63
*aexT*	**26** ^a^	0	**5** ^b^	0	1	0	1	0	1	34
*aexU*	0	0	0	0	0	0	0	0	0	0
*dam*	0	7	**6** ^a^	0	**2** ^b^	0	0	1	0	16
*enolase*	25	32	4	4	2	0	1	1	2	71
*ascV*	0	0	0	0	0	0	0	0	0	0
*hlyA*	0	0	0	0	0	0	0	0	0	0
**Subset of virulence gene**										
*exu*/*ser*/*aer*/*fla*	36	26	5	1	2	0	0	1	4	71
*exu*/*ser*/*aer*	6	9	0	0	0	0	0	0	0	15
*exu*/*aer*/*fla*	2	0	1	0	0	0	0	0	0	3
*exu*/*ser*/*fla*	2	1	0	0	0	0	**1** ^b^	0	0	4
*ser*/*aer*/*fla*	1	0	0	0	0	0	0	0	0	1
*ser*/*fla*	1	0	0	0	0	**2** ^b^	0	0	0	3
*exu*/*fla*	0	0	0	**3** ^a^	0	0	0	0	0	3
*exu* only	0	2	0	0	0	0	0	0	0	2

Significantly higher presence of the single/subset of virulence gene:

^a^ *p*<0.0001;

^b^ *p*<0.05.

Four virulence genes: *exu*, *ser*, *aer* and *fla* were the most frequently detected (>80%) and based on these 4 genes, 8 different subsets were observed and a statistically significant association was observed in *A*. *allosaccharophila* with *exu*/*ser*/*fla*; *A*. *caviae* with *exu*/*fla* and *A*. *media* with *ser*/*fla* (Table 4). The cytotonic enterotoxin genes *alt* and *ast* were less frequently detected (*alt*-7%) and (*ast*-3%) and however mainly present in *A*. *hydrophila*. A significant association (*p*<0.0001) was found between these 2 genes and *A*. *hydrophila* either singly or in combination (*alt*/*ast*), and this implies that *A*. *hydrophila* probably possess a distinct set of virulence genes vis-à-vis other *Aeromonas* species. The *alt* and *ast* virulence determinants are associated with diarrhea, and found to be more prevalent in children with diarrhea compared to healthy controls, probably due to the presence of enterotoxins in *Aeromonas* [19]. The different proportions of virulence genes present suggest that different mechanisms may be used by each subpopulation of *Aeromonas* to colonise and induce infections.

## Conclusions

Phenotypic and genotypic diversity of *Aeromonas* organisms from aquatic environments were investigated in this study. The use of both the approaches are useful for the characterisation of *Aeromonas*, however phenotypic studies have limitations emphasising the need for molecular identification methods. Ninety-six percent of the aeromonad isolates from aquatic environments were confirmed as *Aeromonas* species using the *GCAT* and *rpoD* genes. The results confirm that by the use of these two genes, the definitive identification of environmental *Aeromonas* species is possible. *A*. *veronii* was the predominant species 43%, occurring in the freshwater lakes. Multiple virulence genes were present and different subsets of these genes existed in the various *Aeromonas* species, leading to the suggestion that each species has a distinct set of virulence genes.
